# Inter-laboratory testing of the effect of DNA blocking reagent G2 on DNA extraction from low-biomass clay samples

**DOI:** 10.1038/s41598-018-24082-y

**Published:** 2018-04-09

**Authors:** Carsten Suhr Jacobsen, Tue Kjærgaard Nielsen, Jan Kjølhede Vester, Peter Stougaard, Jeppe Lund Nielsen, Jana Voriskova, Anne Winding, Petr Baldrian, Binbin Liu, Åsa Frostegård, Dorthe Pedersen, Alexander Tøsdal Tveit, Mette Marianne Svenning, Christoph C. Tebbe, Lise Øvreås, Pia Bach Jakobsen, Steven J. Blazewicz, Valerie Hubablek, Stefan Bertilsson, Lars Hestbjerg Hansen, S. Craig Cary, William E. Holben, Flemming Ekelund, Jacob Bælum

**Affiliations:** 1 Department of Environmental Sciences, University of Aarhus, Roskilde, Denmark; 2 Center for Permafrost (CENPERM) and Department of Geochemistry, Geological Survey of Denmark and Greenland, Copenhagen, Denmark; 3 Department of Plant and Environmental Sciences, University of Copenhagen, Frederiksberg, Denmark; 4 Department of Chemistry and Bioscience, Aalborg University, Aalborg, Denmark; 5 Academy of Sciences of the Czech Republic, Bron, Czech Republic; 6 Faculty of Chemistry, Biotechnology and Food Science, Norwegian University of Life Sciences, Ås, Norway; 7 Center for Geomicrobiology, Department of Biosciences, University of Aarhus, Aarhus, Denmark; 8 Department of Arctic and Marine Biology, The Arctic University of Norway, Tromsø, Norway; 9 Thünen-Institut für Biodiversität, Braunschweig, Germany; 10 Department of Biology, University of Bergen, Bergen, Norway; 11 Nuclear and Chemical Sciences Division, Lawrence Livermore National Laboratory, Livermore, CA USA; 12 Department of Ecology and Genetics, Limnology and Science for Life Laboratory, Uppsala University, Uppsala, Sweden; 13 Environmental Research Institute, University of Waikato, Hamilton, New Zealand; 14 Cellular, Molecular and Microbial Biology Program, University of Montana, Missoula, MT USA; 15 Department of Biology, University of Copenhagen, Copenhagen, Denmark; 16 Chr Hansen A/S, Hørsholm, Denmark; 17 Present Address: NovoZymes A/S, Copenhagen, Denmark; 18 Present Address: Berkeley Lab, Berkeley, USA; 19 Present Address: NIRAS A/S, Aarhus, Denmark

## Abstract

Here we show that a commercial blocking reagent (G2) based on modified eukaryotic DNA significantly improved DNA extraction efficiency. We subjected G2 to an inter-laboratory testing, where DNA was extracted from the same clay subsoil using the same batch of kits. The inter-laboratory extraction campaign revealed large variation among the participating laboratories, but the reagent increased the number of PCR-amplified16S rRNA genes recovered from biomass naturally present in the soils by one log unit. An extensive sequencing approach demonstrated that the blocking reagent was free of contaminating DNA, and may therefore also be used in metagenomics studies that require direct sequencing.

## Introduction

Modern microbial ecology studies are often based on extraction of community nucleic acids, followed by molecular analyses of the recovered DNA or RNA. The extreme complexity and variation of the chemical and physical properties of some sample materials such as soils makes it impossible or at least very challenging to standardize nucleic acid extraction procedures. Consequently, numerous different in-house and commercial protocols have been developed and made available for this purpose. In a review, Orgiazzi *et al*.^1^ described the need for a standard across laboratories, and discussed the current ISO standards within this context. Because the ISO standard^2^ is inefficient for extraction of fungal DNA^3^, Orgiazzi *et al*.^1^ recommended a modified protocol; however, this suggestion revealed that the idea of standardization faces obvious problems. Based on these observations we conclude that no single protocol works optimally for all soils and suggest a new modified method targeted for low organic high clay soil.

Clay particles can tightly adsorb organic as well as inorganic phosphorous compounds^4^. Because of the phosphate rich backbone of nucleic acids, these molecules tend to stick to clay sorption sites. Thus, due to their large surface area free clay particles are particularly problematic when extracting the nucleic acids. Essentially, nucleic acids released from lysed cells can be immobilized on particulate adsorption sites before the extraction procedure is completed^5^. Since adsorption is rarely taken into consideration during protocol development, this potential confounder may have a significant influence on the efficiency of DNA extractions from complex matrices, such as soil.

Adsorption of nucleic acids in soil is primarily associated with the inorganic fraction, and especially with minerals such as montmorillonite and kaolinite, which are present at high levels in clay^4–7^. Adsorption in soil increases if the organic fraction is removed, which suggests that the organic fraction has little adsorption potential towards nucleic acids; actually, components of the organic fraction might coat potential nucleic acid binding sites, thereby preventing nucleic acids from binding^8,9^. DNA molecules bound to clay minerals are protected against nucleases and can persist in the environment for a long period of time^6^. Therefore, with regards to extraction of nucleic acids from soil, mineral adsorption introduces two sources of biases: (1) loss of target nucleic acid released upon lysis of microbial cells due to the adsorption, and (2) co-extraction of remaining “ancient” nucleic acids bound in the clay fraction^10^. In protocols for direct DNA and RNA extraction from environmental samples, one parameter of success has been extraction yield, which could be problematic if the aim is to study viable present day organisms. To minimize such contamination by soil-bound nucleic acid, one solution would be to extract and recover cells from soil prior to lysis and DNA extraction although other biases like differential cell lysis may be introduced^11,12^.

Additions of blocking reagents based on skim milk powder, tRNA, or DNA improve DNA extraction efficiency from clay-rich soils^9,13–15^. However, addition of foreign reagents based on biological materials very likely introduces contaminating nucleic acid residues, which may obscure or bias downstream molecular analyses of target nucleic acids. Previously, we developed a blocking reagent (G2) based on short fragments of modified double-stranded DNA, ensuring minimum co-extraction of DNA residues and preventing the added DNA residues from serving as templates for PCR, hybridization, or sequencing^4^. Used in connection with a custom phenol/chloroform-based protocol, the G2 blocking reagent can increase extraction of DNA and mRNA from clay rich groundwater sediments more than 10,000-fold^16^.

The aim of this study was to test the blocking reagent in combination with commercially available DNA extraction kits used for soil in a multiple-lab ring experiment. As the G2 compound is derived from short nucleic acids, we tested whether it contained any contaminating DNA by direct deep sequencing. To test the performance of the blocking reagent in combination with the commonly used MOBIO^TM^ PowerLyzer PowerSoil DNA extraction kit, we performed an inter-laboratory comparison test among 11 different laboratories. All groups received three samples of the same clay sediment and extracted DNA using the same kit, with or without inclusion of the blocking reagent.

## Materials and Methods

### Sampling of soil samples

The clay subsoil used for the multi-laboratory comparison of the extraction protocol was collected at depths between 110 and 130 cm in January 2012 in Kolding, Jutland, Denmark, using handheld drill equipment (Eijkelkamp, Giesbeek, The Netherlands). The intact clay subsoil was kept at 4 °C for 2 months, at which time six replicated 0.25 g portions was weighed out in 2 ml Eppendorf tubes and immediately freeze-dried prior to shipment at ambient temperature to the participating laboratories. The high clay inorganic soil was not homogenized prior to dividing in 0.25 g portions.

### DNA extraction protocol

DNA was extracted from the 0.25 g soil samples by participating laboratories using the protocol provided with the PowerLyzer PowerSoil DNA extraction kit (MOBIO, Carlsbad, CA, USA). From each sample, three replicate samples were subjected to bead-beating using regular 0.1 mm glass beads, and three other replicates from the same sample were subjected to bead-beating using the same 0.1 mm glass beads but pre-coated with lyophilized G2 DNA/RNA enhancer (Ampliqon A/S, Odense, Denmark). The G2 is released from the glass-beads upon mixture with the lysing buffer from the DNA extraction kit and become fully sorbed to the clay within one minute (data not shown).

### Quantitative PCR protocol

The quantitative PCR (q-PCR) reactions were performed in one lab on all samples. Standards with known numbers of 16 S rRNA genes were produced by extraction of DNA from 100 μl of 10-fold dilutions of *E*. *coli* cells washed in 0.015 M phosphate buffer (pH 7.4) using the MOBIO kit. Quantitative PCR reactions were performed in triplicate on all DNA samples using the following setup: 10 µl SsoFast™ EvaGreen® Supermix (BIO-RAD, Hercules, CA, USA), 3.4 µl PCR-grade water (MOBIO, Carlsbad, CA, USA), 400 nM (final concentration) of each primer (341 F: CCTACGGGAGGCAGCAG and 518 R: ATTACCGCGGCTGCTGG)^17^, and 5 µl of 10X diluted template DNA, all in a 20 µl volume. All qPCR preparations were performed on the epMotion 5070 pipetting robot (Eppendorf, Hamburg, Germany) in a high-pressure clean room. qPCR was performed on the CFX96 Touch™ Real-Time PCR Detection System (BIO-RAD) under the following conditions: initial denaturation at 95 °C for 2 minutes; 50 cycles of denaturation at 95 °C for 30 seconds, annealing at 60 °C for 30 seconds, elongation at 72 °C for 45 seconds, and (to prevent quantification of possible primer-dimers) fluorescence measurement at 82 °C for 10 seconds; followed by a final elongation step at 72 °C for 6 minutes.

### DNA loss during extraction protocol and quality control of G2 beads

*Escherichia coli* was grown to near late log phase, and 100,000 dpm ^3^H-thymidine (Sigma-Aldrich, Copenhagen, Denmark) was added in late log phase, while the culture was allowed to continue growing and incorporating the ^3^H-thymidine (the late addition maximizes the amount of ^3^H that is incorporated into DNA). Twenty-five µl of the culture was added to 250 mg of soil and immediately three replicate DNA extractions were performed using the regular PowerLyzer PowerSoil kit (MOBIO, Carlsbad, CA, USA), and three using G2 modified bead tubes (Ampliqon, Odense, Denmark) but otherwise following the exact same protocol. One aliquot of the ^3^H labeled culture and one 10% (vol/vol) aliquot of the DNA extraction were withdrawn at four steps in the protocol (MOBIO): (1) after step 7 (lysis); (2) after step 11 (first inhibitor removal precipitation); (3) after step 14 (second inhibitor removal precipitation); and, finally, (4) after step 23 (final step). The ^3^H signal in the samples was determined by liquid scientilation by combining 0.1 ml of the supernatant with 4 ml of scintillation fluid (Wallac Scintillation Products, Turku, Finland) followed by a 10 mins counting in a liquid scintillation counter (Wallac 1409). Subsequent quality control of G2 beads was performed by following the protocol described above and measuring radioactivity after step 7.

### HiSeq sequencing of potential DNA contamination from G2

*Cupriavidus pinatubonensis* JMP134 was inoculated in Luria–Bertania broth (Alpha BioScience, Baltimore, MD, USA) and incubated with shaking for 24 hours at 28 °C. DNA was extracted from the 2 ml culture both with and without the addition of the G2 compound using the PowerLyzer PowerSoil DNA Isolation Kit (MOBIO). A single-end Illumina HiSeq sequencing library was prepared using a modified version of the NEBNext ® DNA library Prep Master Mix Set kit (New England BioLabs, MA, USA). Briefly, 20 µl DNA was mixed in a PCR tube with 2.4 µl NEBNext 10X Repair Reaction Buffer and 1.25 µl NEBNext End Repair Enzyme which was incubated for 30 minutes at 30 °C. The reaction was purified on a MinElute column (Qiagen, Hilden, Germany) and eluted in 18 µl EB buffer at 37 °C for 15 minutes. In a new PCR tube, the following were added to 17 µl purified DNA: 5 µl Quick Ligation 5X buffer, 0.5 µl of 25 µM stock DNA adaptors for Illumina HiSeq, and 2.5 µl Quick T4 DNA ligase. The reaction was incubated for 15 minutes at 20 °C, then purified on a MinElute column and eluted in 22 µl EB buffer at 37 °C for 15 minutes. In a new PCR tube, the 22 µl of purified DNA was mixed with 2.5 µl NEBNext Adapter Fill-in Reaction Buffer and 1.5 µl *Bst* DNA polymerase. The reaction was incubated for 20 minutes at 65 °C and subsequently heat inactivated at 80 °C for 20 minutes. The resulting DNA library was sequenced on the Illumina HiSeq platform.

Applying the BWA MEM algorithm with default settings^18^, reads were mapped to the genome of *Salmo salar* (RefSeq accession# GCF_000233375.1), as well as to the genome of *Cupriavidus pinatubonensis* JMP134 (RefSeq accession# GCF_000203875.1). Mapping results were explored and the best hit (in case a read mapped to both reference genomes) was determined using Samtools view.

### Statistical analyses

We log-transformed data prior to analysis and then used a mixed ANOVA-model in SAS Enterprise Guide (Ver. 6.1), with addition of G2 as a fixed effect and laboratory as a random effect.

### Importance

Nucleic acid extractions from low-biomass samples are often problematic because (1) the samples contain low levels of DNA and RNA in the first place, and (2) the sample matrix may contain high levels of surfaces with the capacity to adsorp DNA or RNA released from microorganisms following lysis. The DNA/RNA enhancing substance “G2” significantly increased DNA extraction from a low biomass clay subsoil in a multi-laboratory trial and can hence circumvent this difficulty.

### Disclosure

Jacob Bælum and Carsten Suhr Jacobsen are inventors of G2, and according to Danish law the patent was issue by their former employer, The Geological Survey of Denmark and Greenland (GEUS). GEUS later sold the patent to Ampliqon A/S (Odense, Denmark), which produces G2 today. The inventors (JB and CSJ) receive a percentage of the net sale of G2.

## Results and Discussion

### Quantitative-PCR on DNA from inter-laboratory test

Extraction of soil DNA of sufficient quality for applications in molecular biology was pioneered by the Tiedje laboratory^11^, and the protocol by Griffiths *et al*.^19^ has over the years been the leading protocol for co-extracting DNA and RNA from soil samples. The Griffith protocol, however, has serious drawbacks when extracting DNA and RNA from mineral soils that are high in clay and low in organic material; e.g. subsurface soils or groundwater sediment, likely because the clay particles are highly adsorptive^9^. Addition of various nucleic acids or proteins (e.g., salmon sperm DNA, skim milk powder, or tRNA) can overcome problems related to DNA/RNA-adsorbing surfaces^9,14,15,20^.

The inter-laboratory test of the effect of G2 on recovery of DNA from the same high-clay soil using qPCR revealed that G2 consistently increased the 16 S rRNA gene copy number when used in connection with the DNA extraction protocol. Yield increased on an average 7.5 fold and16S rRNA gene copy numbers were significantly (F = 48.8; P < 0.0001) higher in G2-supplemented DNA extractions.

**Figure 1:**
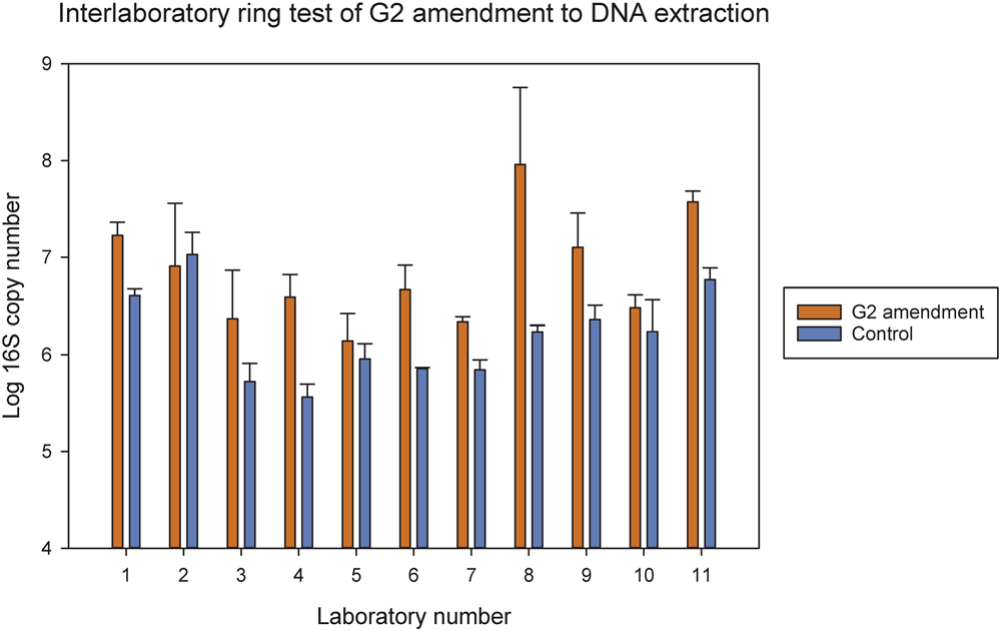
Log copy number of 16S rRNA genes found in one clay subsoil tested in 11 different laboratories using MOBIO PowerLyzer PowerSoil DNA extraction kit with or without G2 coated onto the beads.

The result from this inter-laboratory comparison (Fig. 1) also revealed large differences between the participating laboratories, especially in terms of overall yield of DNA. Today, many molecular microbial ecology laboratories use commercial kits for DNA and RNA extractions for environmental matrices due to their ease of use and expected reproducibility. However, we found that DNA yields differed enormously across participating laboratories, even when they were using the same kit. Using exactly the same soil and exactly the same standard kit (no G2 addition), one laboratory were able to extract around 10^7^ copies of 16 S rRNA genes per gram of soil, whereas others laboratories only obtained fewer than 10^6^ copies. Two main reasons for this high discrepancy can be mentioned – one is that clearly some variation is expected in biomass between the triplicate soil samples, but also some difference in individual skills working with the kit exist, i.e. some laboratories have never tried the kit before and others are using it as a standard DNA extraction method. However, the reason is beyond the scope of this paper, but certainly merits further attention.

### DNA contamination from G2

Addition of a eukaryotic DNA or RNA into a DNA-RNA extraction protocol is only feasible if the additional nucleic acid does not interfere with the purpose of the study, e.g., if the PCR target is a specific bacterial gene, like a respiratory gene^16^, a herbicide degradation gene^21^, or 16 S rRNA genes^9^. In all of these cases, salmon sperm DNA did not lead to false positive results because gene-specific qPCR analysis targeting prokaryotic DNA was used. In theory the addition of DNA or RNA as blocking reagents could be eliminated, if the nucleic acid extraction protocol was gene-specific, using a complementary DNA strand bound to a magnetic particle as hybridization probe fishing out the gene of interest at high stringency conditions^22^.

Metagenomic and metatranscriptomic studies are increasingly becoming part of the standard tool box for many studies within soil microbial ecology. In this context, contaminating DNA is unacceptable. Analysis of 82 and 53 million HiSeq reads of *C*. *pinatubonensis* JMP134 DNA with or without addition of G2 showed in both cases that the same amount of reads had the best match to the standard genome. The reads not mapping to *C*. *pinatubonensis* had similar mapping ratios obtained in the presence and absence of G2, which indicates that no traces of the G2 origin DNA were present in the final DNA extract. Thus, our in-depth sequencing showed no traces of DNA left in the G2 compound. Hence, to our knowledge, this is the only commercially available blocking reagent that can be used to increase yield from low-biomass samples without introducing contaminating DNA.

### Loss of radioactivity during DNA extraction protocol

**Figure 2:**
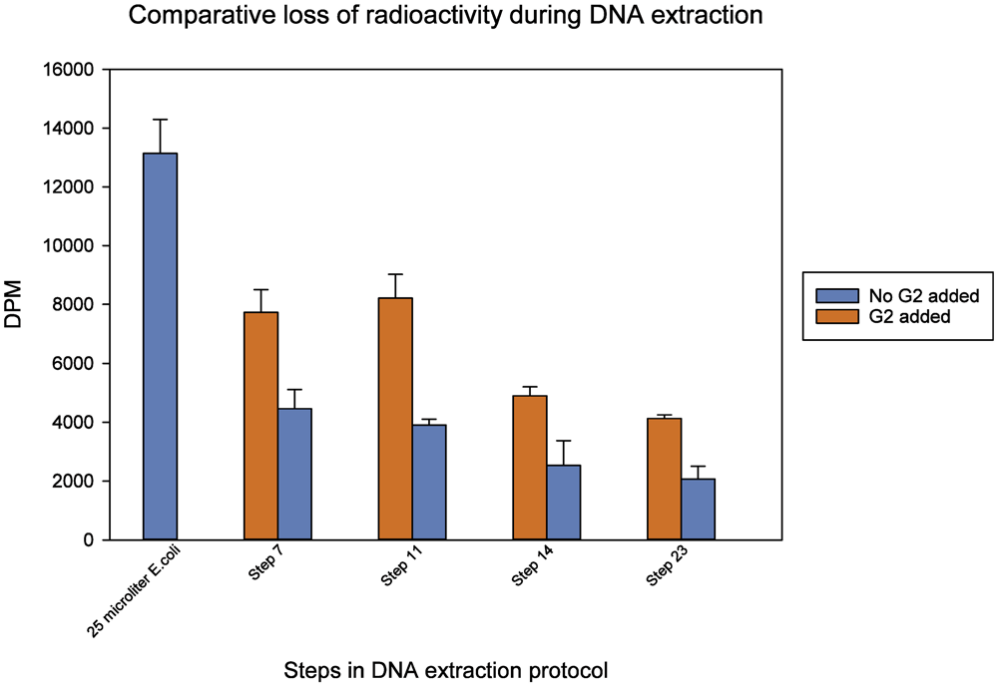
Radioactivity measured at different steps in the MOBIO PowerLyzer PowerSoil DNA extraction procedure. Radioactivity was measured after step 7: supernatant of bead-beated soil, 11: supernatant after removing non-DNA organic and inorganic material including humic substances, cell debris and proteins, 14: supernatant after second removal of non-DNA organic and inorganic material, and 23: final DNA elution.

Batch-tests of G2 beads using thymidine-labeled *E*. *coli* added to the subsurface clay soil showed that about one-third of the radioactivity was lost during the initial steps of the lysis process (MOBIO kit protocol step 7) in the presence of G2, whereas two-thirds of the radioactivity was lost if no G2 was added to the soil (Fig. 2).

Moreover, the protocol steps 11–14 decreased the recovered radioactivity to about half of what was recovered at step 7; finally, at the end of the protocol (step 23), the amount of recovered radioactivity did not differ significantly from the amount recovered at step 14. Furthermore, the ratio between radioactivity recovered with and without addition of G2 remained constant throughout the extraction protocol, i.e., about twice as much radioactivity was recovered in the presence of G2, suggesting that also protein yield is increased by the presence of G2. For soil nucleic acid analyses, the increased presence of such proteins would not be a problem due to the nucleic acid purification procedures applied.
